# An 8-Tap CMOS Lock-In Pixel Image Sensor for Short-Pulse Time-of-Flight Measurements

**DOI:** 10.3390/s20041040

**Published:** 2020-02-14

**Authors:** Yuya Shirakawa, Keita Yasutomi, Keiichiro Kagawa, Satoshi Aoyama, Shoji Kawahito

**Affiliations:** 1 Graduate School of Medical Photonics, Shizuoka University, Hamamatsu 432-8011, Japan; yshira@idl.rie.shizuoka.ac.jp; 2 Research Institute of Electronics, Shizuoka University, Hamamatsu 432-8011, Japan; kyasu@idl.rie.shizuoka.ac.jp (K.Y.); kagawa@idl.rie.shizuoka.ac.jp (K.K.); 3 Brookman Technology, Inc., Hamamatsu 430-0936, Japan; saoyama@brookmantech.com

**Keywords:** CMOS image sensor (CIS), lock-in pixel, short-pulse, time-of-flight (TOF), multi tap, indirect TOF, depth sensing

## Abstract

An 8-tap CMOS lock-in pixel image sensor that has seven carrier-capturing and a draining time window was developed for short-pulse time-of-flight (TOF) measurements. The proposed pixel for the short-pulse TOF measurements has seven consecutive time-gating windows, each of which has the width of 6 ns, which is advantageous for high-resolution range imaging, particularly for relatively longer distances (>5 m) and under high ambient light operations. In order to enhance the depth resolution, a technique for the depth-adaptive time-gating-number assignment (DATA) for the short-pulse TOF measurement is proposed. A prototype of the 8-tap CMOS lock-in pixel image sensor is implemented with a 1POLY 4METAL 0.11-μm CIS process. The maximum non-linearity error of 1.56%FS for the range of 1–6.4 m and the depth resolution of 6.4 mm was obtained at 6.2 m using the DATA technique.

## 1. Introduction

Indirect time-of-flight (TOF) range image sensors are increasingly demanded for various application areas. The application includes smart devices, augmented reality (AR)/virtual reality (VR) systems, security systems, drones, robots, slow vehicles, automated carriers, and automobiles. In order to expand the application areas of TOF imagers, improvements for depth resolution under aggressive conditions, such as longer distance and stronger ambient light, are necessary. Indirect TOF image sensors are classified into two types: a continuous-wave (CW) TOF method [1,2,3,4,5,6,7,8,9] and a short-pulse (SP) TOF method [10,11,12,13,14,15]. In CW-TOF imagers, the high depth resolution and longer distance measurements are simultaneously realized by using higher and multiple (2 or 3) modulation frequencies [5,8]. On the other hand, in the SP-TOF method, the depth resolution is improved by making the gating-time width shorter, and the distance is extended by increasing the number of signal taps of the pixel and by using range-shifting techniques [14,15,16]. For the outdoor use of TOF sensors, the SP-TOF method is more advantageous because a lower amount of ambient light charge is accumulated by using a small-duty, energy-concentrated light pulse and the short gating-time window of the pixel. This results in a high tolerance to the saturation of the pixel and less ambient light shot noise. In addition to this, the pulse-based method has a flexibility regarding the gating clock patterns to be programmed for having advanced functions and performances. Though the reported SP-TOF pixels have maximally four taps for signal outputs [14,15], a SP-TOF pixel with a further number of signal taps will create opportunities of indirect TOF imagers to be used for a variety of applications under aggressive operating conditions.

This paper presents an 8-tap lock-in pixel image sensor [17,18] for SP-TOF measurements. The pixel has eight symmetrical modulation gates, eight charge storage diodes (SDs) with respective transfer gates, and readout source follower amplifiers. The pixel has a function of programming where one of the 8-tap nodes is used for time-gated charge draining and other seven taps are used for time-gated signal outputs. In order to increase the full well capacitance (FWC) and to assist complete charge transfer from the SDs to floating diffusions (FDs), a charge-transfer assisting (CA) gate is implemented in each SD. In Shirakawa et al. [18], a basic operation of the 8-tap lock-in pixels for TOF measurements is shown. The present paper reports a new SP-TOF measurement technique using the 8-tap lock-in pixels and the depth-adaptive time-gating-number assignment (DATA) technique is used, where the number of gating times for the 7-tap outputs are adaptively changed to the distance by considering the dependency of the attenuated reflected light to the distance. Having this technique exploit the feature of flexible programming of gating pulse patterns in SP-TOF method is very effective for obtaining relatively high depth resolution for the entire measurement range by assigning larger gating numbers at farther regions, while suppressing the saturation of signals in the nearest regions by assigning fewer gating numbers. Using this technique, the linearity and range resolution of the 7-tap TOF measurements and TOF range image samples are evaluated.

The rest of this paper is organized as follows. In Section 2, the operation of the multiple-tap lock-in pixel and the distance calculation algorithm are described. Section 3 provides the design of the proposed 8-tap lock-in pixel structure using device simulations. The experimental results of the implemented CMOS 8-tap chip are described in Section 4. Section 5 presents the conclusion.

## 2. Multiple-Tap Lock-In-Pixel and Short-Pulse TOF Measurements

### 2.1. Operation Timing of Multiple-Tap Lock-In-Pixel TOF Imagers

**Figure 1:**
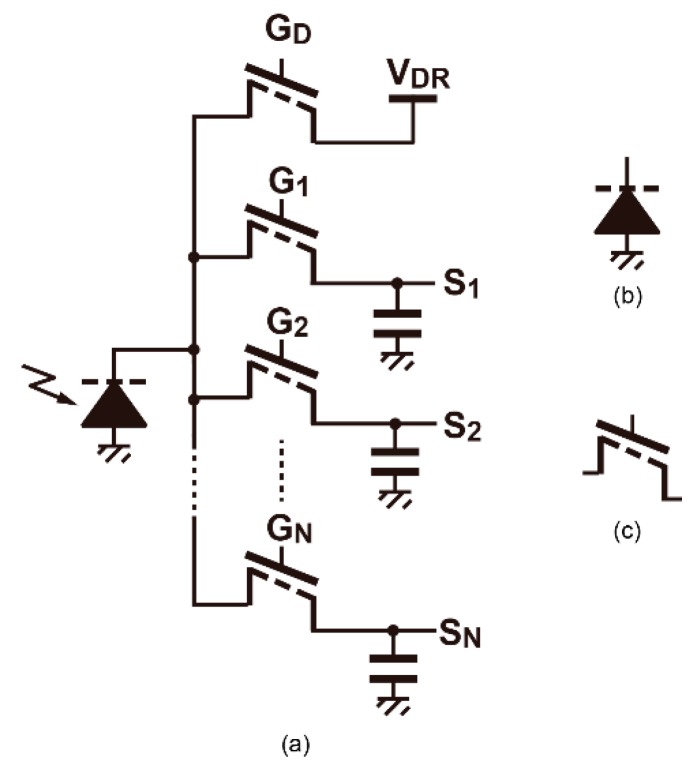
Conceptual schematic of multiple-tap lock-in pixels: (**a**) N-tap lock-in pixel with a drain, (**b**) symbol for a depleted photodiode, and (**c**) symbol for a modulation gate.

**Figure 2:**
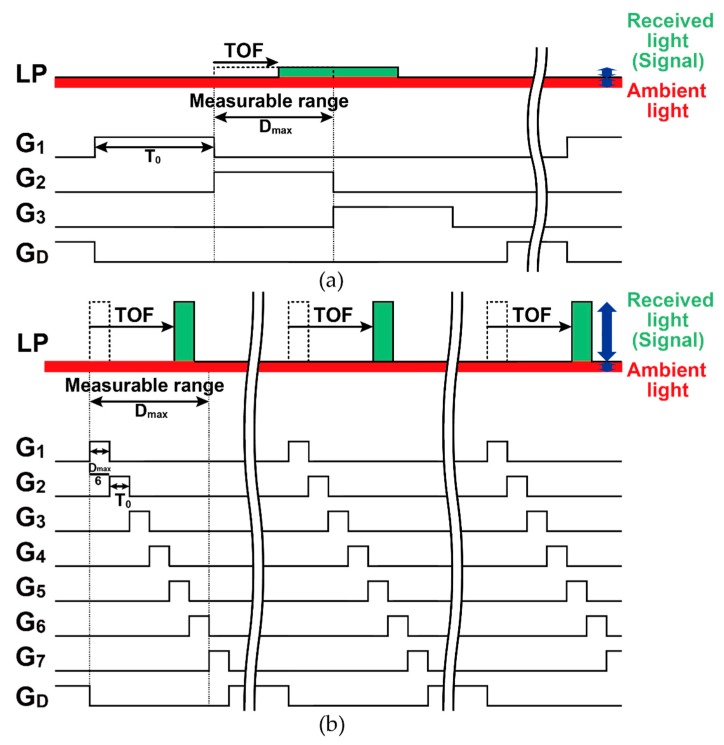
Short-pulse time-of-flight (SP-TOF) measurement using a multiple-tap lock-in pixel imager: (**a**) 3-tap with one window and (**b**) 7-tap with six windows.

Figure 1 shows the conceptual schematic of multiple-tap lock-in pixels for short-pulse TOF measurements and symbols of the parts. The detailed readout circuits used in the pixel are shown in Figure 6a, though these are not shown in Figure 1 for simplicity. The essential part of the pixel consists of a depleted diode, multiple modulation gates, charge storages (capacitors), and a drain. In short-pulse TOF measurement systems, the light is modulated as a train of light pulses and the reflected light from an object is captured by the TOF camera. The operation timing diagrams for two types of multiple-tap lock-in pixels using a short light pulse (LP) are shown in Figure 2. In the indirect TOF method using short-pulse modulation, the generated signal charge created by receiving the reflected light is transferred and shared by two capacitors, and the time-of-flight is measured by calculating the ratio of one of the two charges to the sum of them. For the 3-tap pixel, the TOF is often measured within the range of one time-gating window, as shown in Figure 2a [12,13], using two gated ($G_{2}$, $G_{3}$) signals as TOF-dependent signals and another gated signal ($G_{1}$) for the ambient light charge component. The TOF arrow starts from the $G_{2}$ rising edge in this case because the TOF is calculated using the ratio between charges collected by $G_{2}$ and $G_{3}$. Using the pixel of this work with 7 signal taps and a drain [18], the depth ranges of six time-gating windows can be measured as shown in the timing diagram of Figure 2b. With the assumption that the TOF measurable range (*D_max_*) and the average light (= “peak power” × “duty cycle ratio of light pulse”) for these two cases are the same, a shorter and more energy-concentrated light pulse can be used when increasing the number of signal taps in the lock-in pixels. This property is particularly effective for the operation of the TOF imager under strong ambient light. By increasing the number of signal taps and reducing the width of the resulting time-gating window, the ratio of the signal light to the ambient light intensity is increased and the depth resolution (depth noise) is greatly improved. The depth resolution of the SP-TOF imagers is proportional to the light pulse width (= time-gating width) and the signal-to-noise ratio (SNR). For the operation under no influence from the ambient light and the condition that the photon shot noise of the signal light is dominant for the SNR, the 7-tap pixel with six gating-time windows has a sixfold better depth resolution when compared with that of the 3-tap pixel with a single time-gating window. This is because of the use of the one-sixth shorter light pulse that can be used, and the SNR is unchanged. For the operation under the large influence of ambient light, where the SNR is determined by the shot noise of ambient light, the 7-tap pixel with six time-gating windows has one-sixth of the ambient light charge and $1/\sqrt{6}$ of the ambient light shot noise compared with the 3-tap pixel with a single time-gating window. Therefore, theoretically, the 7-tap pixel with six gating-time windows has $6\times \sqrt{6}\approx 14.7$ times better depth resolution when compared with the 3-tap counterpart.

### 2.2. Depth-Adaptive Time-Gating-Number Assignment for SP-TOF Measurements

In an indirect TOF measurement system, a light source mounted on a TOF camera emits light radially to the 3D measurement space. Then, the intensity of light decreases in a manner that is inversely proportional to the square of the distance from the TOF camera when the target object is assumed to have the perfectly diffusing surface. As a result, the depth resolution becomes the worst at the farthest distance because of the weak signal intensity, while a signal saturation may occur at the closest distance. To address this problem, an efficient SP-TOF measurement technique can be realized using a multiple-tap lock-in pixel imager.

**Figure 3:**
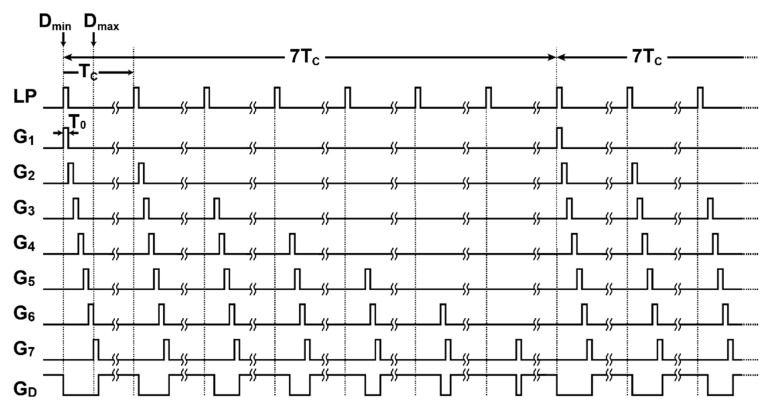
Depth-adaptive time-gating-number assignment for efficient SP-TOF measurement.

Figure 3 shows the SP-TOF measurement timing diagram for considering the dependency of the attenuated reflected light on the distance. In the operation shown in Figure 2b, the gating pulses from *G*_1_ through *G*_7_ are applied consecutively for the closest through to the farthest TOF measurements, respectively. Because of this, the gated signals for the closer objects tend to be too strong, while the gated signals for farther objects tend to be too weak to measure the TOF precisely. In Figure 3, with seven light-pulse cycles as a unit period, the number of gating times for *G*_1_ through *G*_7_ is gradually and adaptively increased into the distance: once per seven cycles for *G*_1_, twice per seven cycles for *G*_2_, and so on. With this operation, named the depth-adaptive time-gating-number assignment (DATA), the amount of signal charge at the farthest gated signal of *G*_7_ is maximized while avoiding the saturation in the closest gated signals of *G*_1_.

### 2.3. Distance Calculation Algorithm

**Figure 4:**
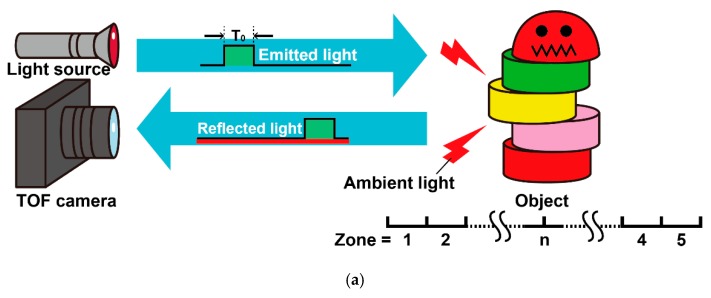

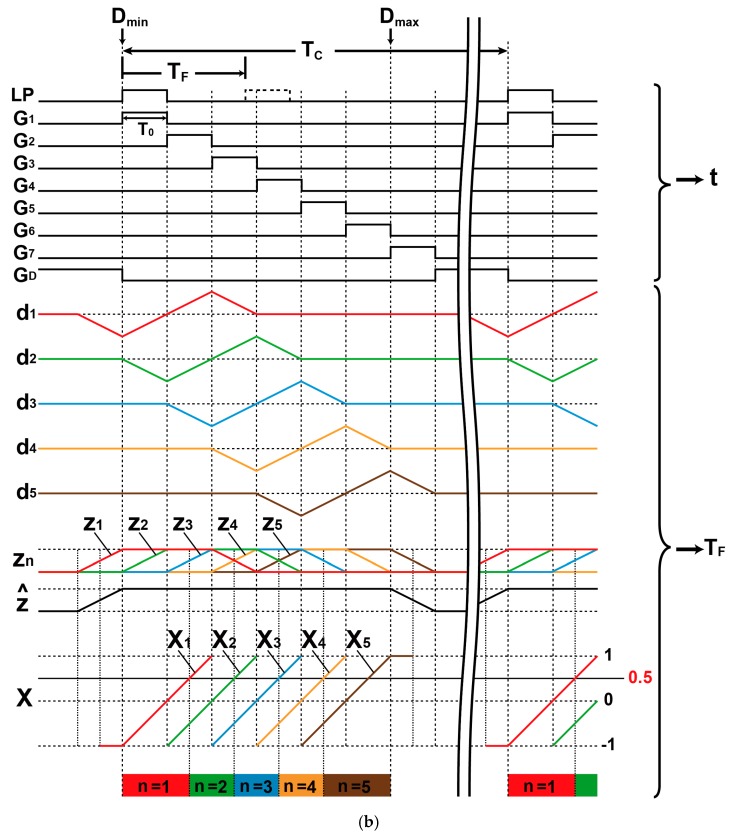
TOF operation of a 7-tap pixel with six time-gating windows: (**a**) principle of a short-pulse TOF measurement system and (**b**) diagram for the distance calculation algorithm.

Figure 4 shows the timing diagram of gating pulses and the corresponding responses to the TOF of a short pulse with the pulse width of $T_{0}$ for the 7-tap lock-in pixel CMOS imager. The light pulse and the gating pulses of the TOF pixel are repeated with the cycle time of $T_{C}$. When the time of flight of the light pulse, $T_{F}$, is varied from zero to the maximum time (7 × $T_{0}$), the generated charge acquired by the nth time-gating window, $q_{n}$, responds as a triangular shape. Then, the difference between the *n*th and the (*n* + 2)th gated charge is expressed as:

(1) $$d_{n}=q_{n+2}-q_{n},$$

which responds linearly to $T_{F}$ from $n\times T_{0}$ to $\left(n+2\right)\times T_{0}$, as shown in Figure 4, where *n* = 1, 2, 3, 4, or 5. If the light pulse is coming when the gates *G*_n+2_ and *G*_n+1_ are activated, the signal charges, *q*_*n*+2_ and *q*_*n*+1_ are generated. If there is an ambient light, *q*_*n*+2_ and *q*_*n*+1_ may also contain ambient light and *q_n_* contains an ambient light charge only. Therefore, Equation (1) describes the cancellation of ambient light charge. Because the light pulse and gating pulses are applied repeatedly with the cycle of $T_{C}$, the behavior of *d*_1_ for the range of *T_F_* < 0 in Figure 4 is considered the same as that for the TOF of *T_C_* + *T_F_*, 2T*_C_* + *T_F_*, 3*T_C_* + *T_F_*, and so on. These five curves as a function of $T_{F}$ are used for measuring the TOF of the back-reflected light. To do this, the variables as a function of $T_{F}$, i.e., $X_{n}$ (*n* = 1, 2, …, 5) shown in Figure 4, is defined as:

(2) $$X_{n}=d_{n}/z_{n},$$

where $z_{n}$ is the signal amplitude estimated using $q_{n}$, $q_{n+1}$, and $q_{n+2}$, and is expressed as:

(3) $$z_{n}=\{\begin{array}{c} q_{n}+q_{n+1}-2q_{n+2}\\ q_{n+1}+q_{n+2}-2q_{n} \end{array}\begin{array}{c} \left(q_{n}-q_{n+2}>0\right)\\ \left(q_{n}-q_{n+2}\leq 0\right) \end{array}.$$

$X_{n}$ responds linearly to $T_{F}$ from $n\times T_{0}$ to $\left(n+2\right)\times T_{0}$ and takes a value from −1 to 1. A normalized signal amplitude $Z_{n}$ is defined as $Z_{n}=z_{n}/\hat{z}$ where $\hat{z}$ is the total amount of the generated charges due to the signal light pulse only, and is expressed as:

(4) $$\hat{z}=\left|q_{3}-q_{1}\right|+q_{2}+q_{4}+q_{6}+\left|q_{7}-q_{5}\right|-\frac{3}{2}\left(\left|q_{7}+q_{5}\right|+\left|q_{7}-q_{5}\right|\right).$$

The response of $z_{n}$ and $\hat{z}$ to the distance is also shown in Figure 4. To choose one of the five curves ($X_{n}$, (*n* = 1, 2, …, 5)) for distance measurements, the flags $F_{n}$ (*n* = 1, 2, …, 5) are defined as:

(5) $$F_{n}=\{\begin{array}{c} “1”\\ “0” \end{array}\begin{array}{c} \left(X_{n}\leq 0.5\right)\\ \left(X_{n}>0.5\right) \end{array};$$

for showing whether X_n_ is larger than 0.5, and the flags $E_{n}$ (*n* = 1, 2, …, 5) are defined as:

(6) $$E_{n}=\{\begin{array}{c} “1”\\ “0” \end{array}\begin{array}{c} \left(Z_{n}>T_{Z}\right)\\ \left(Z_{n}\leq T_{Z}\right) \end{array}$$

for showing whether Z_n_ is larger than $T_{Z}$, where $T_{Z}$ is the threshold for choosing the distance zone. Then, the resulting distance to be measured, *D*, is expressed as:

(7) $$D=\frac{c}{2}T_{0}\left(n+X_{n}\right),$$

where *c* is the velocity of light and *n* is the zone number determined using:

(8) $$n=\{\begin{array}{cc} 1 & \left(\mathrm{if}\;L_{1}=“1”\right)\\ 2 & \left(\mathrm{if}\;L_{2}\cdot \bar{L_{1}}=“1”\right)\\ 3 & \left(\mathrm{if}\;L_{3}\cdot \bar{L_{2}}\cdot \bar{L_{1}}=“1”\right)\\ 4 & \left(\mathrm{if}\;L_{4}\cdot \bar{L_{3}}\cdot \bar{L_{2}}\cdot \bar{L_{1}}=“1”\right)\\ 5 & \left(\mathrm{if}\;\bar{L_{4}}\cdot \bar{L_{3}}\cdot \bar{L_{2}}\cdot \bar{L_{1}}=“1”\right) \end{array},$$

where $L_{n}$ (*n* = 1, 2, 3, 4) are logical variables defined as $L_{n}=F_{n}\cdot E_{n}$.

## 3. Design of an 8-Tap Lock-In Pixel Image Sensor for SP-TOF Measurements

### 3.1. Pixel Structure

**Figure 5:**
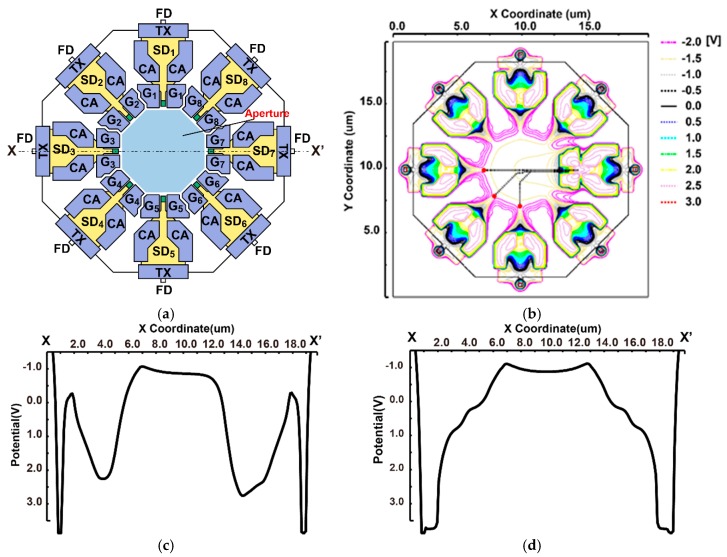
3D device simulation results: (**a**) structure of the 8-tap lock-in pixel, (**b**) charge transportation result, (**c**) potential diagram of the storage diode (SD) accumulation mode (X–X’), and (**d**) potential diagram for the charge transfer mode from the SD to floating diffusion (FD) (X–X’).

Figure 5a shows the structure (top view) of the designed 8-tap lock-in pixel. A pinned photodiode (PPD) as a photo detector is located in the central region, and the flow of photo-charges to eight directions is controlled by eight modulation gates concentrically placed around the PPD. The modulation gates ($G_{1}$ through $G_{8}$) are implemented using a lateral electric field charge modulation (LEFM) [19]. The modulated charge at each tap is stored in a storage diode (SD) [20,21]. The SD basically has a structure of a pinned diode, and while having a sufficient amount of full-well capacitance (FWC) and helping the charge transfer from the SD to the floating diffusion (FD), a charge-transfer assisting (CA) gate is used for each tap [17]. During the charge transfer from the PPD to the SD, the potential well voltage of the SD is raised by the CA gate being biased to a high voltage (2.3 V). When reading the charge by transferring the accumulated charge in the SD to the FD by the transfer (TX) gate, the potential well depth of the SD is lowered by the CA gate to be biased to a low voltage (−1.0 V). This operation assists in transferring the charge from the SD to the FD while preventing backward charge retransfer from the FD to the SD.

In this pixel, three n-type doping layers are used under the p-type layer for the PPD. The first n-type layer is used entirely for the PPD, LEFM gates, and SDs; the second n-layer is mainly used for the LEFM and the SDs; and the third n-layer is used mainly for the SDs. This triple n-doping helps us to create depleted potential profiles to accelerate photo carriers from the PPD to the selected SDs (one of eight SDs) activated by one of the eight LEFM gates.

Figure 5b,c shows the 3D device simulation results when the generated photo-carriers are to be transferred to $SD_{7}$. The LEFM gate $G_{7}$ and CA gates are biased to a high voltage (2.3 V) and the other LEFM gates ($G_{1}$ through $G_{6}$ and $G_{8}$) are biased to a low voltage (−2 V). It can be seen that a potential gradient is generated in the photo-sensing area to transfer photo-carriers to the $SD_{7}$, as shown in Figure 5c. In Figure 5b, red points denote the initial positions (depth: 3 μm) of an electron generated by a photon and black dotted lines indicate the movement trace of an electron. The transfer time of carriers from the initial position to the $SD_{7}$, which is calculated by the velocity of carriers as a function of the electric field, is 990 ps.

Figure 5d shows the potential diagrams along with the X–X’ lines shown in Figure 5a when the accumulated carriers in the SD are transferred to the FD. In this case, the TX gates are biased to a high voltage (3.3 V) and the LEFM gates ($G_{1}$ through $G_{8}$) and CA gates are biased to a low voltage (LEFM gate: −2 V, CA gate: −1 V). As can be seen from this result, the channel potentials around $SD_{1}$ and $SD_{7}$ are relatively low and these potentials prevent the charges from transferring to the untargeted SDs, while the CA gate creates a large potential gradient, which attracts carriers to be transferred to the FD.

### 3.2. Pixel Circuits and Readout Operations

**Figure 6:**
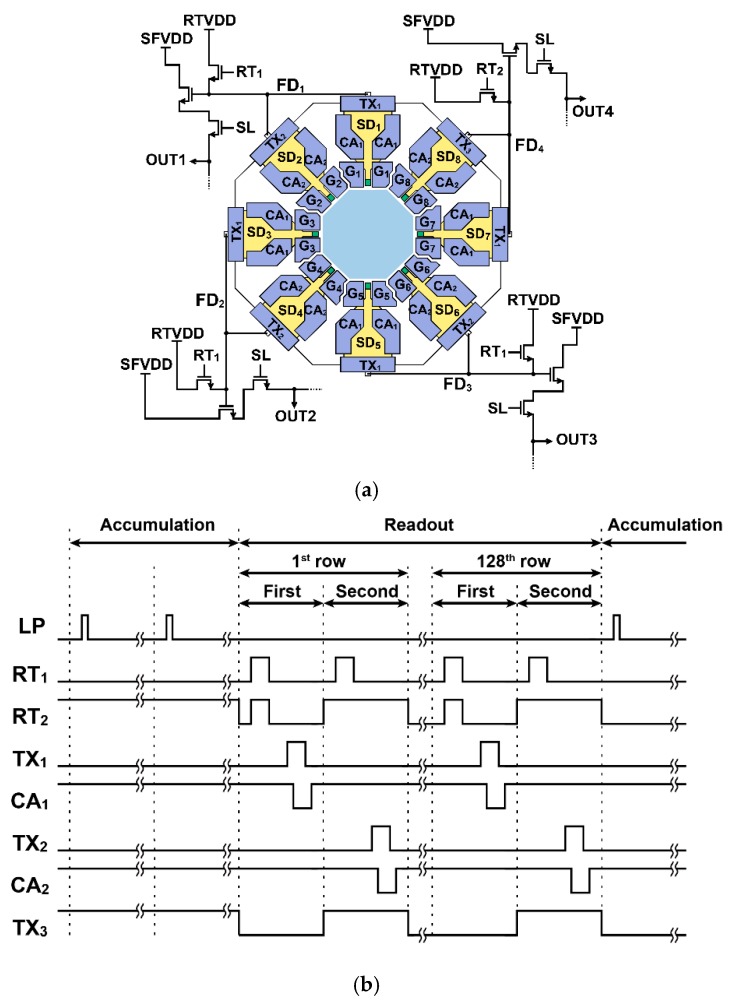
Pixel circuits and readout operation: (**a**) equivalent circuit and (**b**) timing diagram for accumulating and reading photo signals.

Figure 6a,b shows the pixel schematic with readout circuits and a timing diagram for accumulating and reading photo signals in each pixel. The pixel has four parallel outputs, and four source follower amplifiers are shared with two adjacent SDs to save the in-pixel circuit area. As shown in Figure 6b, the TOF image acquisition cycle, or frame period, is divided into an accumulation phase and a readout phase. In the accumulation phase, the eight modulation gates are driven as shown in the timing of Figure 2b or Figure 3. After the accumulation phase, the readout phase is started. The readout process from the pixel array is done on a row-by-row basis. To read out all the accumulated signals in the SDs, the readout operation cycle is repeated twice. In the first cycle, the floating diffusion node is reset using reset gates ($RT_{1}$ and $RT_{2}$), then the $TX_{1}$ gate is turned on for reading out the accumulated signals in $SD_{1}$, $SD_{3}$, $SD_{5}$, and $SD_{7}$. For assisting the charge transfer from the SDs, the CA gate is used. The action of the CA gates is a little delayed (about 10 ns) from the turn-on and turn-off timings of the TX gate. In the second cycle, the floating diffusion node is reset using $RT_{1}$; then, the $TX_{2}$ gate is turned on for reading out the accumulated signals in $SD_{2}$, $SD_{4}$, and $SD_{6}$. In the pixel operation for reading 7-tap signal outputs, the node for $SD_{8}$ is used for draining such that the $FD_{4}$ node is always reset by setting $RT_{2}$ to high during the second cycle. The four signals from the pixel are read out using analog CDS (correlated double sampling) circuits at the column and are converted to digital signals using column analog-to-digital converters (ADCs).

## 4. Implementation and Experimental Results

### 4.1. Implemented TOF Imager Chip

**Figure 7:**
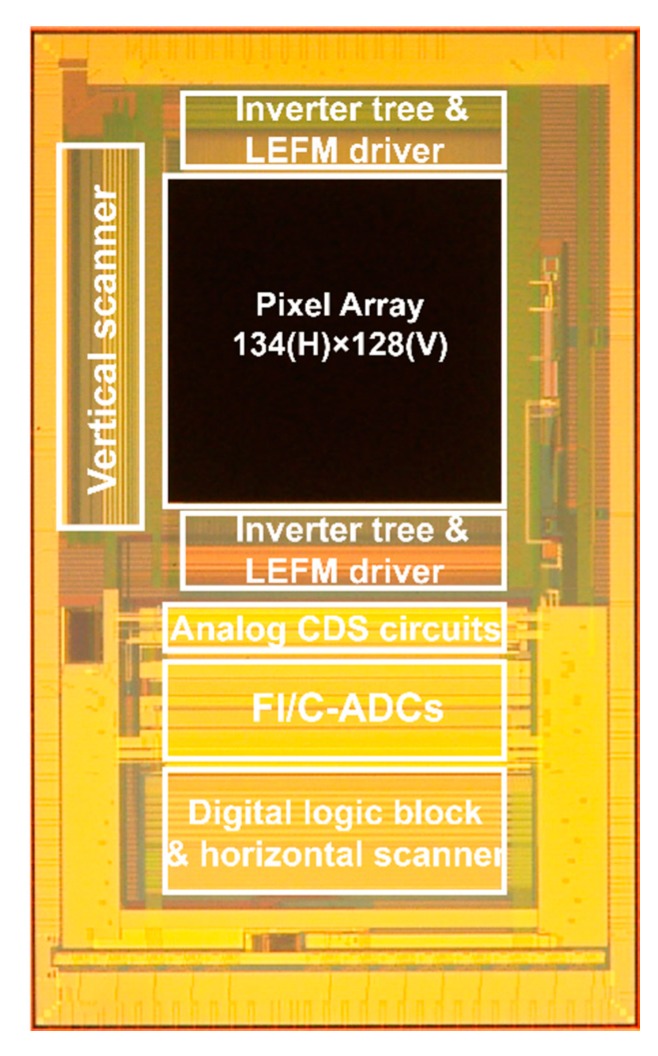
Chip micrograph. CDS: correlated double sampling, FI/C-ADC: Folding-Integration/Cyclic ADC, LEFM: lateral electric field charge modulation.

Figure 7 shows a photo micrograph of a prototype 8-tap lock-in-pixel TOF imager chip implemented using a 1Poly 4Metal 0.11-μm CMOS image sensor process. The pixel array had a total of 134(H) × 128(V) pixels, including test pixels. An inverter tree and a driver for pixel gating were arranged above and below the pixel array for faster gate responses. The pixel outputs were read out by using a 19-bit column ADC [22]. The converted digital signal was horizontally read out using low-voltage differential signaling (LVDS) circuits. Table 1 shows a brief summary of the prototype TOF imager specification and basic characteristics. The chip was assembled to the cooling package, had Peltier elements, and was cooled down to roughly −15 °C to suppress the influence of the dark current in the measurements. The relatively large dark current in the present design was related to the structure around the CA gate, which must be improved for further low-noise performance of the chip. The readout time of 51.84 ms was very long for the imager given the relatively small number of pixels. One reason for this long readout time was the use of a multiple-sampling based column ADC [22]. The implemented chip had a non-linearity problem if the operation clock of the ADC was increased. To reduce the non-linearity, the following measurement data were taken with a slower clock for the ADC than that of the designed specification. The readout speed could be improved by re-designing the column ADC of the chip.

### 4.2. Charge Modulation Characteristics

**Figure 8:**
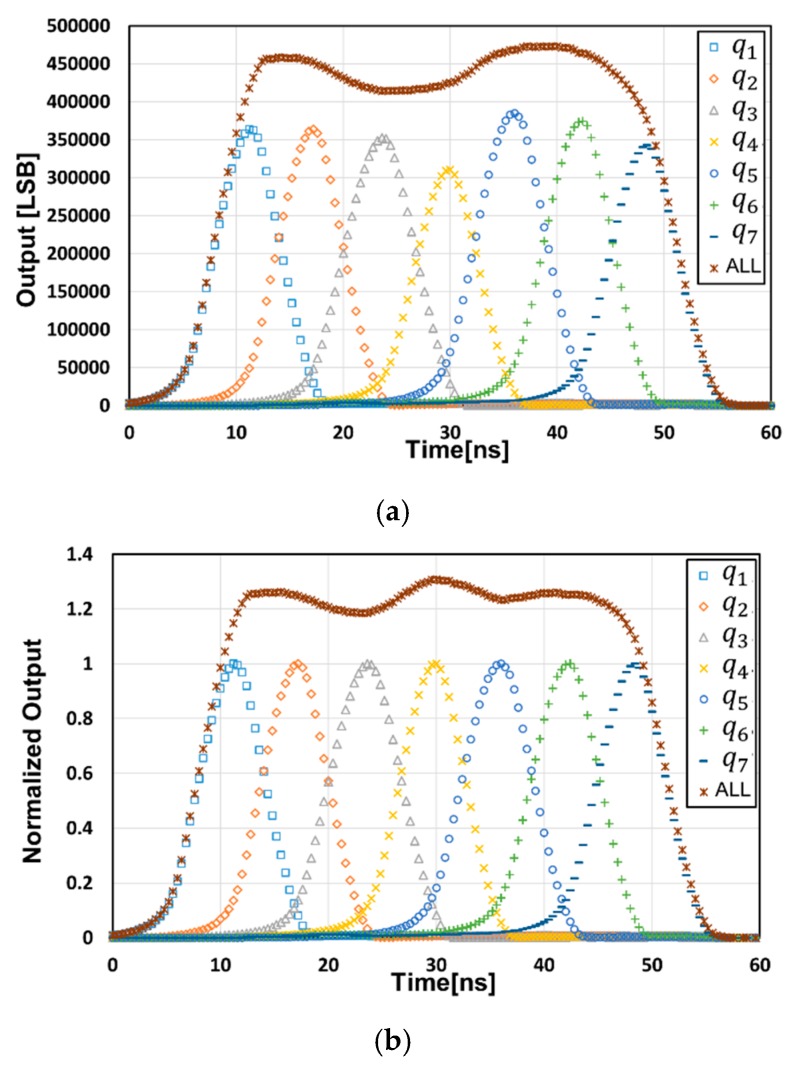
Response of the seven signal-tap outputs to the light pulse delay: (**a**) measured raw data and (**b**) the normalized data.

**Figure 9:**
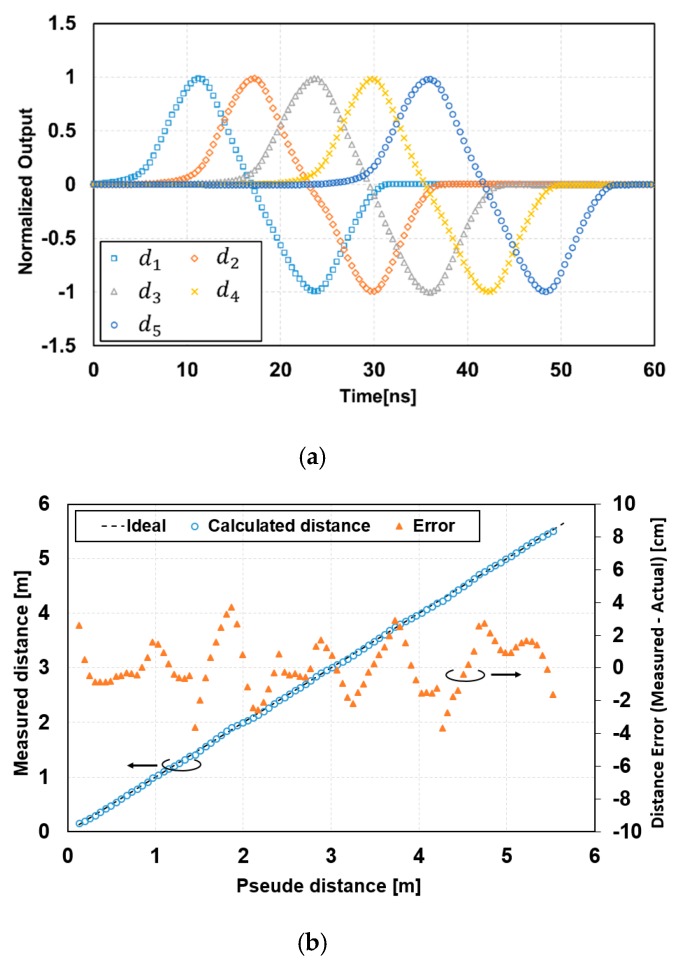
Differential responses and calculated distances using the normalized response curves: (**a**) differences of two signal-tap outputs and (**b**) calculated distance with seven signal-tap outputs versus the pseudo distance.

Figure 8 shows a measurement result of the response of seven signal-tap outputs to the light pulse delay. A laser pulse with a pulse width of 6 ns, a wavelength of 850 nm, a power of 29 mW/m^2^ (0.88 W/m^2^ peak) at 6 m and a laser pulse rate of 5.5 MHz was used. A lens with a large focal length of 12.5 mm was used For the sensor characterization, the sensor field-of-view (FOV) used for the measurement was 13.1°(V) × 13.7°(H). In this measurement, the modulation gates, *G*_1_ through *G*_7_, were opened as shown in the timing of Figure 2b with *T*_0_ = 6 ns, and the delay of the laser pulse was scanned from 0 to 60 ns with a step of 0.4 ns. The gate *G*_8_ was used as a charge draining gate and the $SD_{8}$ was working as a drain. Figure 8a shows measured data after subtracting the dark signal from the raw data and Figure 8b shows a normalized result with a sensitivity correction. The average demodulation contrast in the charge signals stored in SDs, for $q_{1}$ through $q_{7}$, was 0.83. As shown in Figure 8, the response of the seven signals to the pulse delay time was fast enough for the short-pulse TOF measurement with the light pulse width of 6 ns when compared with the ideal response (triangular shape), where it was rising linearly over 6 ns and falling linearly over 6 ns. Using the normalized response of Figure 8b, the differences of the two signal-tap outputs ($d_{1}$, $d_{2}$, $d_{3}$, $d_{4}$,$\;d_{5}$) were calculated and are shown in Figure 9a. The five piecewise-linear response curves, two of which overlap each other, were obtained and these were used for the TOF range measurements by switching these five curves properly, as described in Section 2.3. Figure 9b shows a curve for the calculated (measured) distance, i.e., the pseudo distance, which corresponded to twice the TOF. This linearity plot was obtained using a calibration applying the gain and offset corrections to the five piecewise-linear response curves. After this non-linearity correction, the maximum of the measured distance error was 3.7 cm and the maximum non-linearity error was 0.67%FS in the range of 0 m to 5.4 m.

### 4.3. Distance Measurements

**Figure 10:**
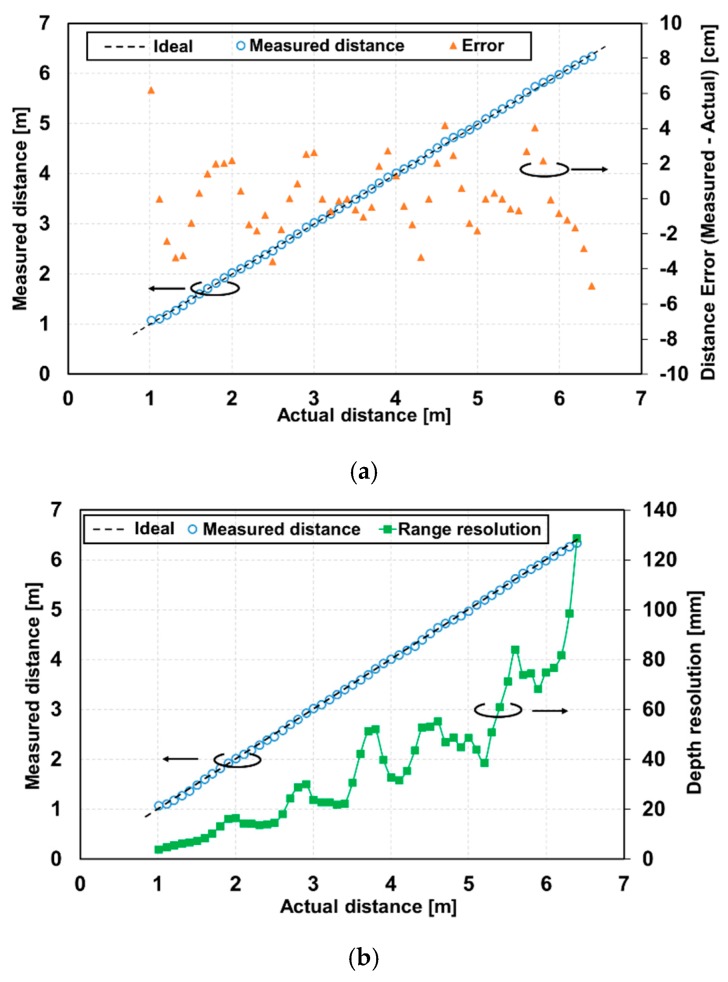
Measured distance versus real distance with modulation timing being the same accumulation time: (**a**) non-linearity error and measured distance, and (**b**) depth resolution and measured distance.

Using a similar calculation method for the TOF described in Section 4.2, distance measurements were carried out with two types of gating methods, as shown in Figure 2b (ordinary time-gating method) and Figure 3 (depth-adaptive time-gating-number assignment (DATA) method). The emitted laser pulse width and pixel-gating pulse width were 6 ns. The measured object was a white panel with the reflection factor of 99% and a fully diffusing surface. The white panel was moved with a 0.1 m step from 1.0 m to 6.4 m from the TOF camera. The range linearity and depth resolution were measured using 100 frames. In the ordinary gating method, the accumulation time in all SDs was set to 0.217 ms. This setting of a short accumulation time was necessary to avoid the saturation in the SDs that received a strong light power due to the reflections with very close objects. The SD_1_ was the most critical and the SD_2_ the second most critical. Figure 10 shows the distance measurement results of the non-linearity error (Figure 10a) and depth resolution (Figure 10b).

The pixel area of 11(H) × 11(V) is used for the measurements. The threshold $T_{Z}$ for calculating the distance used in Equation (6) was set to 0.5. The maximum measured distance error was 6.2 cm and the maximum non-linearity error was 1.15%FS for the full range of 1–6.4 m. For the case of excluding the near end of the measurement range, the measured distance error was less than 5.4 cm, which means the non-linearity error was less than 1%FS. The depth resolution in this method was less than 20 mm up to 2.6 m, and it increased to 128 mm at the maximum distance. The degradation of the depth resolution with increasing distance was due to the attenuated reflected light and the limited FWC of the pixel.

**Figure 11:**
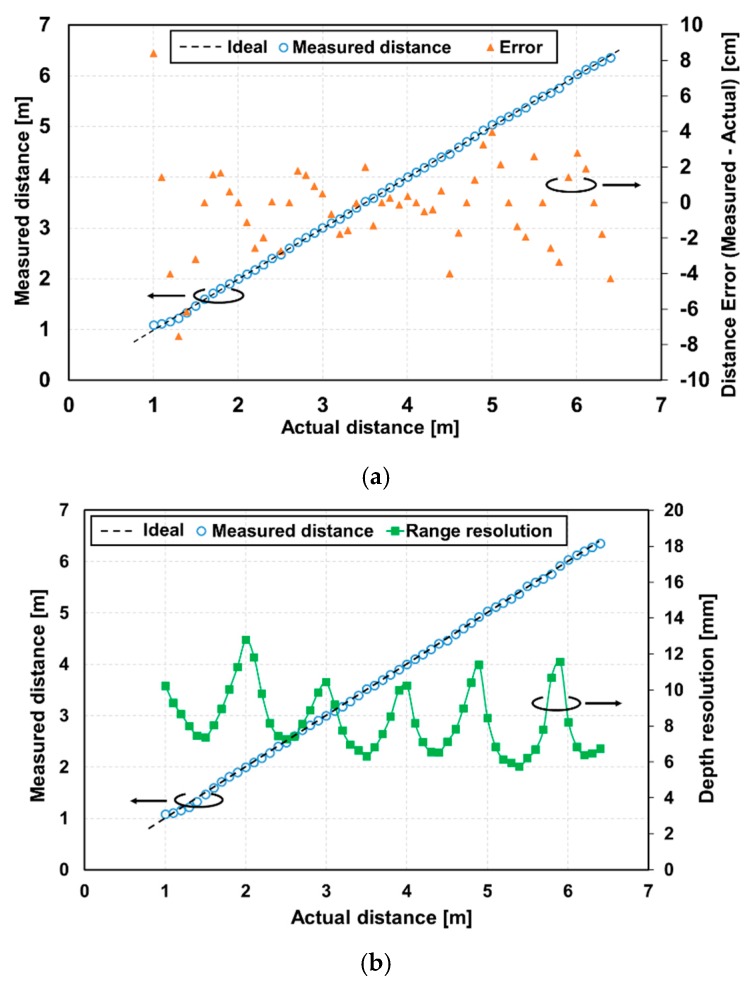
Measured distance versus real distance with an SP-TOF-specific timing: (**a**) non-linearity error and measured distance, and (**b**) depth resolution and measured distance.

Figure 11 shows the distance measurement results using the DATA method shown in Figure 3. In this method, the accumulation times in $SD_{1}$ through $SD_{7}$ were 0.169 ms, 0.384 ms, 1.008 ms, 1.794 ms, 2.187 ms, 3.760 ms, and 4.153 ms, respectively. The pixel area of 11(H) × 11(V) was used for the measurements. The maximum of the measured distance error was 8.4 cm and the maximum non-linearity error was 1.56%FS for the full range of 1–6.4 m. For the measurement ranges larger than 1.5 m, the measured distance error was less than 5.4 cm, which means the non-linearity error was less than 1%FS, as shown in Figure 11a. Figure 11b shows the depth resolution as a function of the distance.

Using the DATA method, where the optimum accumulation time was assigned depending on the reflected light intensity, a good depth resolution smaller than 13 mm was obtained for the entire measurement range and the resolution of 6.4 mm was obtained at 6.2 m. The depth resolution corresponded to 0.10% of the depth.

**Figure 12:**
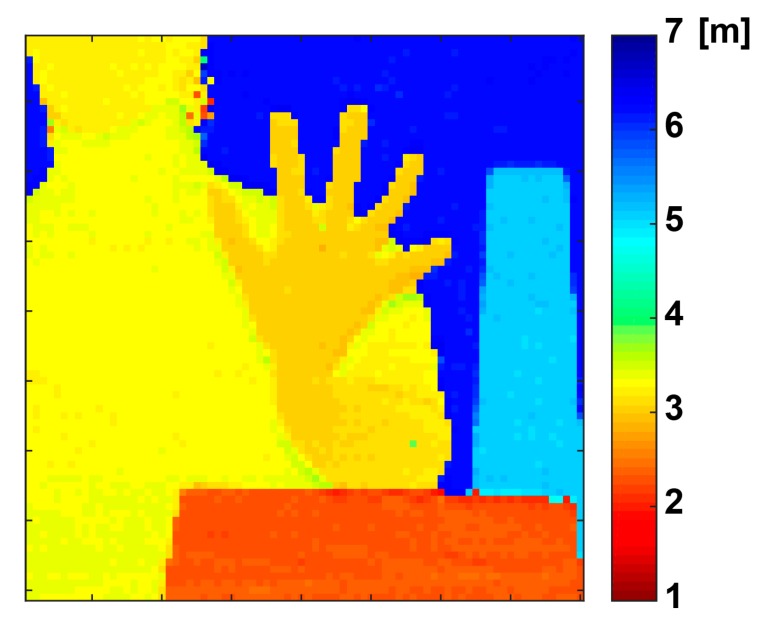
Depth image.

Figure 12 shows a depth image with the implemented TOF image sensors using the operation of the depth-adaptive time-gating-number assignment technique. This depth image with only the subset (80(H) × 81(V) pixels) of the entire pixel array (134(H) × 128(V)) is shown because of the relatively small irradiating angle of the laser light used. In the scene, a white box was placed at 2.1 m and 4.7 m, a manikin was placed at 3.1 m, and the white screen was placed at 6.0 m as a background. The threshold $T_{Z}$ for calculating the distance used in Equation (6) was set to 0.3. This image was generated by using one frame of data.

## 5. Conclusions

This paper presents an 8-tap CMOS lock-in pixel image sensor for short-pulse TOF measurements. The proposed operation of the 8-tap lock-in pixel image sensor using seven taps for signal outputs and a tap for draining was suitable for high-resolution, single-frame TOF measurements using small-duty short light pulses. The implemented prototype had a good response to a short light pulses, where a pulse width of 6 ns was used for TOF measurements. The pulse-based TOF measurements with multiple-tap pixels allowed us to use a flexible time-gating pattern setting. The proposed technique with depth adaptive time-gating-number assignment was effective for realizing a wide measurement range and a high depth resolution. Using this technique and the 8-tap pixel image sensor, the high depth resolution of 6.4 mm at 6.2 m (0.10% of depth) and better than 13 mm within whole depth range of 1 m to 6.4 m were obtained. Because of the segmented TOF measurements with seven time-gating windows, the linearity was also relatively good. For the case of pseudo distance measurements using a delay generator for pseudo TOF generation, a maximum non-linearity error of 0.67%FS was demonstrated for the distance range from 0 m to 5.4 m. Distance calculation algorithms consecutively using the 7-tap outputs were developed and depth images covering 1 m to 6.4 m were successfully obtained.

## Figures and Tables

**Table 1:** Brief summary of the prototype CMOS imager performance. CIS: CMOS image sensor.

Parameter	Value
Technology	0.11-μm 1P4M CIS process
# of pixels	134 (H) × 128(V)
Pixel size	22.4 μm × 22.4 μm
Chip size	5874 μm × 9342 μm
ADC resolution	19 bit
Readout time	51.84 ms
Conversion gain	69.5 $\mu V/e^{-}$
Full well capacity	5600 $e^{-}$
